# Supplementary material for: Mental health interventions for suicide prevention among indigenous adolescents: a systematic review protocol
Source: BMJ Open. 2020 May 17;10(5):e034055. doi: 10.1136/bmjopen-2019-034055 (PMC7239512; doi:10.1136/bmjopen-2019-034055)
Supplement: Author's manuscript [file bmjopen-2019-034055.draft_revisions.pdf]

BMJ Open

BMJ Open is committed to open peer review. As part of this commitment we make the peer review history of every article we publish publicly available.

When an article is published we post the peer reviewers' comments and the authors' responses online. We also post the versions of the paper that were used during peer review. These are the versions that the peer review comments apply to.

The versions of the paper that follow are the versions that were submitted during the peer review process. They are not the versions of record or the final published versions. They should not be cited or distributed as the published version of this manuscript.

BMJ Open is an open access journal and the full, final, typeset and author-corrected version of record of the manuscript is available on our site with no access controls, subscription charges or pay-per-view fees (http://bmjopen.bmj.com).

If you have any questions on BMJ Open's open peer review process please email info.bmjopen@bmj.com

BMJ Open

Mental health interventions for suicide prevention among indigenous adolescents: a systematic review protocol

Journal:	*BMJ Open*
Manuscript ID	bmjopen-2019-034055
Article Type:	Protocol
Date Submitted by the Author:	03-Sep-2019

Complete List of Authors:	Grande, Antonio José ; Mato Grosso State University Medical Course<br>Elia, Christelle; King's College London Faculty of Life Sciences and Medicine<br>Peixoto, Clayton; Federal University of Rio de Janeiro<br>Jardim, Paulo de Tarso Coelho; State University of Mato Grosso do Sul<br>Dazzan, Paola; King's College London Faculty of Life Sciences and Medicine<br>Veras, Andre Barciela; State University of Mato Grosso do Sul<br>Cruickshank, John; King's College London Faculty of Life Sciences and Medicine<br>Harding, Seeromanie; King's College London Faculty of Life Sciences and Medicine

Keywords:	Suicide & self-harm < PSYCHIATRY, Adolescent, Indigenous, MENTAL HEALTH, Child & adolescent psychiatry < PSYCHIATRY, PUBLIC HEALTH

SCHOLARONE™
Manuscripts

I, the Submitting Author has the right to grant and does grant on behalf of all authors of the Work (as defined in the below author licence), an exclusive licence and/or a non-exclusive licence for contributions from authors who are: i) UK Crown employees; ii) where BMJ has agreed a CC-BY licence shall apply, and/or iii) in accordance with the terms applicable for US Federal Government officers or employees acting as part of their official duties; on a worldwide, perpetual, irrevocable, royalty-free basis to BMJ Publishing Group Ltd ("BMJ") its licensees and where the relevant Journal is co-owned by BMJ to the co-owners of the Journal, to publish the Work in this journal and any other BMJ products and to exploit all rights, as set out in our licence.

The Submitting Author accepts and understands that any supply made under these terms is made by BMJ to the Submitting Author unless you are acting as an employee on behalf of your employer or a postgraduate student of an affiliated institution which is paying any applicable article publishing charge ("APC") for Open Access articles. Where the Submitting Author wishes to make the Work available on an Open Access basis (and intends to pay the relevant APC), the terms of reuse of such Open Access shall be governed by a Creative Commons licence – details of these licences and which Creative Commons licence will apply to this Work are set out in our licence referred to above.

Other than as permitted in any relevant BMJ Author's Self Archiving Policies, I confirm this Work has not been accepted for publication elsewhere, is not being considered for publication elsewhere and does not duplicate material already published. I confirm all authors consent to publication of this Work and authorise the granting of this licence.

For peer review only

Title page

Title: Mental health interventions for suicide prevention among indigenous adolescents: a systematic review protocol

Authors:

Antonio Jose Grande[1] 0000-0001-7182-075, Christelle Elia[2], Clayton Peixoto[3], Paulo de Tarso Coelho Jardim[1], Paola Dazzan[4], Andre Barciela Veras[1], J Kennedy Cruickshank[5], Seeromanie Harding[2]

[1]Medical Course, State University of Mato Grosso do Sul, Campo Grande, Mato Grosso do Sul, Brazil,CEP 79115-898, Antonio Jose Grande adjunct professor, Paolo de Tarso Coelho adjunct professor

[2]Department of Nutritional Science, School of Life Course Sciences, Faculty of Life Sciences & Medicine, King's College London, Franklin Wilkins Building, London SE1 9NH, UK, Christelle Elia research assistant, Seeromanie Harding professor in social epidemiology

[3] Institute of Psychiatry, Federal University of Rio de Janeiro (UFRJ), Rio de Janeiro, Brazil CEP 22290-140 Clayton Peixoto PhD candidate

[4]Institute of Psychiatry, Psychology and Neuroscience, Kings College London, Denmark Hill Campus, London SE5 9RJ, UK, Paola Dazzan professor of neurobiology of psychosis

[5]Cardiovascular Medicine & Social Epidemiology group, Division of Diabetes & Nutritional Sciences, Kings College London, Franklin Wilkins Building, London SE1 9NH, UK, J Kennedy Cruickshank professor of cardiovascular medicine & diabetes

Word count: 2083

Corresponding author:
Antonio Jose Grande
Av. Dom Antônio Barbosa, 4155 - Vila Santo Amaro, Campo Grande
Mato Grosso do Sul, Brazil. CEP 79115-898
Phone Number +55 67 34115326
email: grandeto@uems.br

Transparency statement The corresponding author attests that all listed authors meet authorship criteria and that no others meeting the criteria have been omitted.

For peer review only

Title: Mental health interventions for suicide prevention among indigenous adolescents: a systematic review protocol

Abstract

Introduction: There are more the 370 million Indigenous people from 5,000 cultures and living in 90 countries worldwide. Although they make up 5% of the global population, they account for 15% of the extreme poor. Youth suicide is the second leading cause of mortality among 15-29 years old and disproportionately affects indigenous youth. This research protocol for a systematic review will use a comparator/control group to evaluate the effectiveness of suicide interventions targeting indigenous adolescents (aged 10-19 years).

Methods and analysis: We will conduct a systematic search on MEDLINE, EMBASE, CINAHL, LILACS, PsycINFO from inception to September 2019 to identify articles that compare mental health interventions for suicide prevention among indigenous adolescents. Two reviewers independently will determine the eligibility of each study. Studies will be assessed for methodological quality using the risk of bias tool to assess non-randomized studies of interventions. We will conduct a meta-analysis if possible and use established statistical methods to identify and control for heterogeneity where appropriate.

Ethics and dissemination: This systematic review will use published data; thus, ethical permission is not required. Findings will be disseminated through a peer-reviewed publication and will be made available to key decision-makers with authority for indigenous health and other relevant stakeholders.

Registration details: The study protocol is registered at International prospective register of systematic reviews (PROSPERO) ID number 141754.

Keywords: indigenous, adolescents, suicide prevention, mental health, interventions

ARTICLE SUMMARY

Strength and limitations of this study

- Our systematic review will fill a gap in the evidence-base by providing a comprehensive assessment of the existing literature that compare the effectiveness of mental health interventions for suicide prevention among indigenous adolescents.
- Rigorous methods following at least two independent study authors for screening, data extraction and critical appraisal.
- The findings of this systematic review will inform decision-makers about interventions to prevent suicide among indigenous adolescents

For peer review only - http://bmjopen.bmj.com/site/about/guidelines.xhtml

- It is possible to find paucity of data in this field, which would locate a research gap and help plan future studies on the topic.

INTRODUCTION

There are more the 370 million Indigenous people from 5,000 cultures and living in 90 countries worldwide. Although they make up 5% of the global population, they account for 15% of the extreme poor. Despite representing a rich diversity of cultures, they share common concerns and continue to be among the world's most marginalised groups, with historic poorer physical and mental health compared with their non-indigenous counterparts. Life expectancy of indigenous people is about 20 years lower than non-Indigenous[1]. Social exclusion, poverty and socio-environmental change are important contributors to the health disparities across countries and indigeneity causing widespread mental health distress[2]. The effects of colonization and policies of forced assimilation continue to be a cause of intergenerational trauma, manifested through feelings of marginality, depression, anxiety and confusion, which places individuals at increased risk of suicide[3].

Youth suicide is the second leading cause of mortality among 15-29 years old[4] and disproportionately affects indigenous youth[5,6,7]. Indigenous children (5–17 years old) in Australia die from suicide at five times the rate of their non-Indigenous peers (10.1 per 100 000 vs 2 per 100 000 in 2013–17). Similarly, in New Zealand, the suicide rate in Maori youth aged 15–24 years is 40.7 per 100 000 (vs 15.6 per 100 000 in non-Maori youth), and in Inuit youth in Canada the figure is 11 times the non-Indigenous average[8]. Most interpretations of this gap highlight the persistent social disadvantage experienced by indigenous youth relative to non-indigenous youth[6]. The epidemic of youth suicide is relatively recent in some indigenous cultures, appearing in the last 30 to 40 years[9]. Indigenous suicide rates appear to have increased over time, more so in the latter half of the 20th century, with men accounting for the majority of suicides, and the 15-24 years old group having the highest suicide rates of any age group[10]. Furthermore, suicide among indigenous young people may be unreported due to misclassification. Risk factors include mental health disorders, stressful life events, substance abuse, and poor physical health all of which occur at disproportionately higher rates in indigenous populations[11,12]. Suicide in youth is also known to occur in clusters and one of the strongest risk factors is having a friend attempt or commit suicide, and be affected by depression, conduct disorders and substance and alcohol abuse[5]. Protective factors include high support (social and familiar), and cultural connectedness and personality factors such as high self-esteem and internal locus of control and increasing age[5].

Over the last 20 years, Indigenous people's rights have been increasingly recognised through international organisations such as the United Nations Permanent Forum on Indigenous Issues, which has a permanent forum for youth. The 2030 Agenda for Sustainable Development refers to indigenous people 6 times[13]: 3 times in the political declaration; twice in the target under Goal 2 on Zero Hunger (target 2.3) and Goal 4 on education (target 4.5). Many of the Sustainable Development Goals (SDGs) are relevant for indigenous peoples, particularly the focus on reducing inequalities.

For peer review only - http://bmjopen.bmj.com/site/about/guidelines.xhtml

The global indicator framework that measures progress of implementation of the 17 SDGs includes 2 indicators referring directly to indigenous people among several others that are relevant.

With such high rates of suicide among Indigenous youth and considering that youth suicide was virtually non-existent prior to the 1950's[5], culturally tailored suicide interventions are urgently needed. Strategies need to ensure they address the economical, socio-environmental and historical issues that contributed to the emergence of youth suicide in indigenous culture. Two previous systematic reviews examined suicide prevention strategies in indigenous populations[9,10] and one assessed the methodological rigour in indigenous youth suicide prevention studies[5]. Clifford, Doran and Tsey (2013) investigated suicide prevention strategies among indigenous people of all ages. Harlow and Clough (2014) evaluated suicide prevention programmes targeting Indigenous youths until 2012 and had no restriction on study designs. This article presents the protocol for a systematic review of studies which use a comparator/control group to evaluate the effectiveness of suicide interventions targeting indigenous adolescents (aged 10-19 years).

OBJECTIVES

To synthesize the scientific evidence on suicide prevention programmes targeting Indigenous youth

METHODS AND ANALYSIS

This protocol adheres to the Preferred Reporting Items for Systematic reviews and Meta-Analyses Protocols (PRISMA-Equity) guidelines[14]. The study protocol is registered at International prospective register of systematic reviews (PROSPERO) ID number 141754.

Types of studies

We will include any randomised or non-randomised studies, which has a control or comparative group.

Types of participants

The participants are adolescents aged 10-19 years who are self-identified as Indigenous peoples by individuals and acceptance as such by their community. We plan to investigate mental health problems in this population as defined by the DSM-V.

Types of interventions

We will include in person or e-health interventions and which have been delivered to young indigenous people around the world. We will consider a wide range of delivery channels (e.g., in person, online, phone), different practitioners (healthcare practitioners, teachers, lay health care providers) and sectors (i.e., health, primary, secondary and tertiary care, education, guardianship councils).

Types of outcome measures

Primary outcomes

For peer review only - http://bmjopen.bmj.com/site/about/guidelines.xhtml

- Self-injury acts
- Suicidal ideation
- Suicide attempts
- Completed suicide

Secondary outcomes

- Wellbeing / Quality of life

- Social Functioning including Educational Outcomes

Search methods for identification of studies

Electronic searches

We will search the Cochrane Central Register of Controlled Trials (CENTRAL, latest issue, MEDLINE (1966 to latest issue), EMBASE (1974 to latest issue), CINAHL (1981 to latest issue), LILACS (Latin American and Caribbean Health Sciences) (1982 to latest issue), PsycINFO (1887 to latest issue). We will use the search strategies described in Appendix 1 to search MEDLINE and adapt to every other database. We will not use any language or publication restrictions. In case of articles in other languages, we will use academic networks to translate the critical parts to enable screening of abstracts and, if necessary, critical parts of the full paper.

Searching other resources

We will check the reference lists of all primary studies and review articles for additional references. We will email experts in the field about other published and unpublished studies that may be eligible for inclusion.

Inclusion criteria

We will include non-randomised and randomised studies. Electronic database search for theses and dissertations. We will also include studies that used data collection techniques (individual interviews, focus groups and observation) or data analysis processes characteristic of the qualitative method (for example: thematic analysis, content analysis).

We will also consider the following inclusion criteria:

• Self-identification as Indigenous peoples by individuals and acceptance as such by their community
• Historical continuity and land occupation before invasion and colonisation
• Strong links to territories (land and water) and related natural resources
• Distinct social, economic, or political systems
• Distinct language, culture, religion, ceremonies, and beliefs
• Tendency to form non-dominant groups of society
• Resolution to maintain and reproduce ancestral environments and systems as distinct peoples and communities
• Tendency to manage their own affairs separate from centralised state authorities

For peer review only

Exclusion criteria

If the population included in study is not Indigenous or there is no distinction between Indigenous and non-Indigenous populations.

If youth are not included in study or are only briefly mentioned or adult population.

Data collection and analysis

We will work in pairs, independently, for example: Antonio Jose Grande (AJG) and Paulo de Tarso (PT), will independently analyse 1000 titles and abstracts of studies found in the databases. Other researchers will analyse 1000 titles and abstracts, and so on. If necessary, a third reviewer Seeromanie Harding (SH) will be called on to resolve any discrepancies. The results will be imported into EndNoteX8 software.

After both authors have discussed the results of the selection process and have made a consensus decision on which articles to be included/excluded, the data from each of the articles meeting the eligibility criteria will be extracted.

A standardised, pre-piloted form will be used to extract data from the included studies for assessment of study quality and evidence synthesis. Missing data will be requested from study authors. The draft format will include:

Study details: aim, study design, design details, country in which study was conducted, details on location of intervention delivery (i.e., city or community), target condition / risk factor (i.e., subthreshold symptoms, experience of child maltreatment)

Participants: sample size (intervention and control groups at baseline and follow-up), sociodemographic characteristics (e.g., age, gender, ethnicity, socioeconomic status)

Intervention details: description of intervention including frequency and duration of treatments /sessions, mode of delivery (face to face, internet), format (one to one or group), cost of intervention

Delivery of the intervention: setting in which intervention was delivered (school, home, healthcare practice), who delivered the intervention (i.e., medical doctor, nurse, psychologist, teacher, lay health worker etc) and whether it was delivered by one practitioner or a team of individuals or online, whether there was intersectoral collaboration (i.e., between health and education or guardianship councils)

Data extraction and management

We will work in pairs, independently for example: Antonio Jose Grande (AJG) and Paulo de Tarso (PT), will independently extract data.

Assessment of risk of bias in included studies

The methodological quality of the included studies will be evaluated by pairs, independently, for example: Antonio Jose Grande (AJG) and Paulo de Tarso (PT), will independently analyse the included studies. Depending on study design of the included study we will choose the tool to assess the study.

For peer review only

Measures of treatment effect

Types of measurements of treatment effect that may be used:

1. Dichotomous data: we will use risk ratio (RR) for likely binary outcomes, prevalence ratio for some outcomes.

2. Continuous data: we will combine the results using the mean difference (MD) for measures using the same scale or the standardised mean difference (SMD) where different scales have been used to evaluate the same outcome

Unit of analysis issues

We will consider the individual as the unit of analysis. We will separate the non-randomized from randomized studies if we have enough studies for meta-analysis.

Dealing with missing data

We will send two emails (one initial, one reminder) to the corresponding author to ask for any missing data or incompletely reported study details. We will check for consistency between studies and analyse each outcome.

Assessment of heterogeneity

We will assess the inconsistencies between studies using the I2 statistic and describe the percentage of variability in effect. We will consider heterogeneity substantial if I2 is over 50%. See below for subgroup analysis.

Assessment of reporting biases

If mismatches are identified between study protocols and reports, we will contact the trial authors to clarify the information. We plan to explore the impact of including such studies by conducting a sensitivity analysis. We will perform a funnel plot asymmetry test if 10 or more trials are included.

Data synthesis

We will present the data separately for randomized and non-randomized studies. We will meta-analyse trials if the combination of data for the outcomes is possible. We will use a random-effects model, independently of the heterogeneity identified. Forest plot graphics produced by RevMan 5.3 will illustrate meta-analyses. If the combination of data is not possible, we will present a narrative analysis of individual studies. We will create a 'Summary of findings' table using the outcomes proposed in this protocol and we will present the quality of the body of evidence considering the five GRADE assumptions (study limitations, consistency of effect, imprecision, indirectness and publication bias) contributing to the meta-analyses for the pre-specified outcomes.

Subgroup analysis and investigation of heterogeneity

We plan to explore the subsets or subgroups of countries/regions, for primary, secondary or tertiary care, type of mental health condition, gender, age, urban/rural area.

Sensitivity analysis

For peer review only - http://bmjopen.bmj.com/site/about/guidelines.xhtml

We will pool included studies to verify whether the impact of risk of bias affects the overall effect. We will explore which studies increased heterogeneity.

Ethics and dissemination

This systematic review will use published data; therefore, ethical permission is not required. Findings will be published in a peer-reviewed scientific journal. Our results will also be presented at national and international conferences and will be made available to key decision-makers with authority for indigenous health.

Quality of the evidence

Two review authors (AJG, CP) will independently rate the quality of the outcomes. We will use GRADE to rank the quality of the evidence using the GRADEprofiler Guideline Development Tool software[15] and the guidelines provided in Chapter 11 of the Cochrane Handbook for Systematic Reviews of Interventions[16].

CONCLUSION

This review will present the best-available evidence for decision making on suicide prevention programmes targeting indigenous youth in the literature. It will provide in one single document all the primary research on the topic with the quality assessment for each single paper and will rank the quality of evidence produced.

References

1. World Bank (2016) 'Understanding Poverty. Indigenous People', available online https://www.worldbank.org/en/topic/indigenousPeoples (accessed July 17th, 2019).
2. Kirmayer LJ, Fraser SL, Fauras V, Whitley R. Current approaches to aboriginal youth suicide prevention. Cultural and Mental Health Research Unit working paper. 2009;14.
3. Lawson-Te Aho K, Liu JH. Indigenous suicide and colonization: The legacy of violence and the necessity of self-determination. International Journal of Conflict and Violence (IJCV). 2010 May 7;4(1):124-33.
4. Patton GC, Coffey C, Sawyer SM, Viner RM, Haller DM, Bose K, Vos T, Ferguson J, Mathers CD. Global patterns of mortality in young people: a systematic analysis of population health data. The lancet. 2009 Sep 12;374(9693):881-92.
5. Harder HG, Rash J, Holyk T, Jovel E, Harder K. Indigenous youth suicide: a systematic review of the literature. Pimatisiwin: A Journal of Aboriginal and Indigenous Community Health. 2012;10(1):125-42.
6. Hunter E, Harvey D. Indigenous suicide in Australia, New Zealand, Canada and the United States. Emergency Medicine. 2002 Mar;14(1):14-23.
7. Pollock NJ, Naicker K, Loro A, Mulay S, Colman I. Global incidence of suicide among Indigenous peoples: a systematic review. BMC medicine. 2018 Dec;16(1):145.
8. The LC. Suicide in Indigenous youth: an unmitigated crisis. The Lancet. Child & adolescent health. 2019 Mar;3(3):129.
9. Harlow AF, Clough A. A systematic review of evaluated suicide prevention programs targeting indigenous youth. Crisis. 2014.

For peer review only - http://bmjopen.bmj.com/site/about/guidelines.xhtml

10. Clifford AC, Doran CM, Tsey K. A systematic review of suicide prevention interventions targeting indigenous peoples in Australia, United States, Canada and New Zealand. BMC public health. 2013 Dec;13(1):463.
11. Gracey M, King M. Indigenous health part 1: determinants and disease patterns. The Lancet. 2009 Jul 4;374(9683):65-75.
12. King M, Smith A, Gracey M. Indigenous health part 2: the underlying causes of the health gap. The lancet. 2009 Jul 4;374(9683):76-85.
13. United Nations Division for Sustainable Development (2018b) 'Indigenous Peoples and the Post-2015 Development Agenda', available online at https://www.un.org/development/desa/IndigenousPeoples/focus-areas/post-2015agenda.html (accessed May 8, 2019)
14. Welch V, Petticrew M, Tugwell P, Moher D, O'Neill J, Waters E, et al. (2012) PRISMA-Equity 2012 Extension: Reporting Guidelines for Systematic Reviews with a Focus on Health Equity. PLoS Med 9(10): e1001333. doi:10.1371/journal.pmed.1001333
15. GRADEpro Guideline Development Tool [Software] [Computer program]. McMaster University (developed by Evidence Prime, Inc.), Available from www.gradepro.org, 2015.
16. Schünemann HJ, Oxman AD, Higgins JPT, Vist GE, Glasziou P, Akl E, et al, on behalf of the Cochrane GRADEing Methods Group and the Cochrane Statistical Methods Group. Chapter 11: Completing 'Summary of findings' tables and grading the confidence in or quality of the evidence. In: Higgins JPT, Churchill R, Chandler J, Cumpston MS, editor(s). Cochrane Handbook for Systematic Reviews of Interventions version 5.2.0 (updated June 2017). Cochrane, 2017 (available from www.training.cochrane.org/handbook).

Funding statement This work was supported by the Medical Research Council (MRC) grant number MR/R022739/1

Authors' contributions AJG and CE wrote the protocol; PJ, CP, PD, SH critical review the protocol.

Competing interests The authors declare no competing interests

For peer review only - http://bmjopen.bmj.com/site/about/guidelines.xhtml

Appendix 1. Search strategy MEDLINE (PubMed)

#1 Native* OR indigenous OR aboriginal OR ethnic* OR First nations OR population groups

#2 (“mental health” OR Schizophrenia OR Psychosis OR Mania OR Mood OR Internalizing OR Externalizing OR Affective OR Behavioural OR alcohol OR drugs OR “crack cocaine” OR addiction OR “mental illness” OR well-being OR suicide OR depression OR happiness OR wellness OR emotion* OR psych* OR trauma OR stress OR disorder OR psychology)

For peer review only

Reporting checklist for protocol of a systematic review.

Based on the PRISMA-P guidelines.

Instructions to authors

Complete this checklist by entering the page numbers from your manuscript where readers will find each of the items listed below.

Your article may not currently address all the items on the checklist. Please modify your text to include the missing information. If you are certain that an item does not apply, please write "n/a" and provide a short explanation.

Upload your completed checklist as an extra file when you submit to a journal.

In your methods section, say that you used the PRISMA-Preporting guidelines, and cite them as:

Moher D, Shamseer L, Clarke M, Ghersi D, Liberati A, Petticrew M, Shekelle P, Stewart LA. Preferred Reporting Items for Systematic Review and Meta-Analysis Protocols (PRISMA-P) 2015 statement. Syst Rev. 2015;4(1):1.

		Reporting Item	Page Number
**Title**			
Identification	#1a	Identify the report as a protocol of a systematic review	1,2
Update	#1b	If the protocol is for an update of a previous systematic review, identify as such	N/A
**Registration**			
	#2	If registered, provide the name of the registry (such as PROSPERO) and registration number	2
**Authors**			
Contact	#3a	Provide name, institutional affiliation, e-mail address of all protocol authors; provide physical mailing address of corresponding author	1
Contribution	#3b	Describe contributions of protocol authors and identify the guarantor of the review	1,9

For peer review only

**Amendments**			
	#4	If the protocol represents an amendment of a previously completed or published protocol, identify as such and list changes; otherwise, state plan for documenting important protocol amendments	
**Support**			
Sources	#5a	Indicate sources of financial or other support for the review	9
Sponsor	#5b	Provide name for the review funder and / or sponsor	9
Role of sponsor or funder	#5c	Describe roles of funder(s), sponsor(s), and / or institution(s), if any, in developing the protocol	9
**Introduction**			
Rationale	#6	Describe the rationale for the review in the context of what is already known	3,4
Objectives	#7	Provide an explicit statement of the question(s) the review will address with reference to participants, interventions, comparators, and outcomes (PICO)	4
**Methods**			
Eligibility criteria	#8	Specify the study characteristics (such as PICO, study design, setting, time frame) and report characteristics (such as years considered, language, publication status) to be used as criteria for eligibility for the review	4
Information sources	#9	Describe all intended information sources (such as electronic databases, contact with study authors, trial registers or other grey literature sources) with planned dates of coverage	5
Search strategy	#10	Present draft of search strategy to be used for at least one electronic database, including planned limits, such that it could be repeated	Appendix
Study records - data management	#11a	Describe the mechanism(s) that will be used to manage records and data throughout the review	6
Study records - selection process	#11b	State the process that will be used for selecting studies (such as two independent reviewers) through each phase of the review (that is, screening, eligibility and inclusion in meta-analysis)	6

For peer review only

Study records - data collection process	#11c	Describe planned method of extracting data from reports (such as piloting forms, done independently, in duplicate), any processes for obtaining and confirming data from investigators	6
Data items	#12	List and define all variables for which data will be sought (such as PICO items, funding sources), any pre-planned data assumptions and simplifications	6
Outcomes and prioritization	#13	List and define all outcomes for which data will be sought, including prioritization of main and additional outcomes, with rationale	4,5
Risk of bias in individual studies	#14	Describe anticipated methods for assessing risk of bias of individual studies, including whether this will be done at the outcome or study level, or both; state how this information will be used in data synthesis	7
Data synthesis	#15a	Describe criteria under which study data will be quantitatively synthesised	7
Data synthesis	#15b	If data are appropriate for quantitative synthesis, describe planned summary measures, methods of handling data and methods of combining data from studies, including any planned exploration of consistency (such as I2, Kendall's τ)	
Data synthesis	#15c	Describe any proposed additional analyses (such as sensitivity or subgroup analyses, meta-regression)	
Data synthesis	#15d	If quantitative synthesis is not appropriate, describe the type of summary planned	7
Meta-bias(es)	#16	Specify any planned assessment of meta-bias(es) (such as publication bias across studies, selective reporting within studies)	
Confidence in cumulative evidence	#17	Describe how the strength of the body of evidence will be assessed (such as GRADE)	8

None The PRISMA-P checklist is distributed under the terms of the Creative Commons Attribution License CC-BY 4.0. This checklist can be completed online using https://www.goodreports.org/, a tool made by the EQUATOR Network in collaboration with Penelope.ai

For peer review only

BMJ Open

Mental health interventions for suicide prevention among indigenous adolescents: a systematic review protocol

Journal:	*BMJ Open*
Manuscript ID	bmjopen-2019-034055.R1
Article Type:	Protocol
Date Submitted by the Author:	11-Feb-2020
Complete List of Authors:	Grande, Antonio José ; Mato Grosso State University Medical Course<br>Elia, Christelle; King's College London Faculty of Life Sciences and Medicine<br>Peixoto, Clayton; Federal University of Rio de Janeiro<br>Jardim, Paulo de Tarso Coelho; State University of Mato Grosso do Sul<br>Dazzan, Paola; King's College London Faculty of Life Sciences and Medicine<br>Veras, Andre Barciela; State University of Mato Grosso do Sul<br>Cruickshank, John; King's College London Faculty of Life Sciences and Medicine<br>Harding, Seeromanie; King's College London Faculty of Life Sciences and Medicine
**Primary Subject Heading**:	Mental health
Secondary Subject Heading:	Evidence based practice, Epidemiology, Mental health, Global health, Health policy
Keywords:	Suicide & self-harm < PSYCHIATRY, Adolescent, Indigenous, MENTAL HEALTH, Child & adolescent psychiatry < PSYCHIATRY, PUBLIC HEALTH

SCHOLARONE™
Manuscripts

BMJ

I, the Submitting Author has the right to grant and does grant on behalf of all authors of the Work (as defined in the below author licence), an exclusive licence and/or a non-exclusive licence for contributions from authors who are: i) UK Crown employees; ii) where BMJ has agreed a CC-BY licence shall apply, and/or iii) in accordance with the terms applicable for US Federal Government officers or employees acting as part of their official duties; on a worldwide, perpetual, irrevocable, royalty-free basis to BMJ Publishing Group Ltd ("BMJ") its licensees and where the relevant Journal is co-owned by BMJ to the co-owners of the Journal, to publish the Work in this journal and any other BMJ products and to exploit all rights, as set out in our licence.

The Submitting Author accepts and understands that any supply made under these terms is made by BMJ to the Submitting Author unless you are acting as an employee on behalf of your employer or a postgraduate student of an affiliated institution which is paying any applicable article publishing charge ("APC") for Open Access articles. Where the Submitting Author wishes to make the Work available on an Open Access basis (and intends to pay the relevant APC), the terms of reuse of such Open Access shall be governed by a Creative Commons licence – details of these licences and which Creative Commons licence will apply to this Work are set out in our licence referred to above.

Other than as permitted in any relevant BMJ Author's Self Archiving Policies, I confirm this Work has not been accepted for publication elsewhere, is not being considered for publication elsewhere and does not duplicate material already published. I confirm all authors consent to publication of this Work and authorise the granting of this licence.

For peer review only

Title page

Title: Mental health interventions for suicide prevention among Indigenous adolescents: a systematic review protocol

Authors:

Antonio José Grande[1] 0000-0001-7182-075, Christelle Elia[2], Clayton Peixoto[3], Paulo de Tarso Coelho Jardim[1], Paola Dazzan[4], Andre Barciela Veras[1], John Kennedy Cruickshank[5], Seeromanie Harding[2]

[1]Medical Course, State University of Mato Grosso do Sul, Campo Grande, Mato Grosso do Sul, Brazil,CEP 79115-898, Antonio Jose Grande adjunct professor, Paolo de Tarso Coelho adjunct professor

[2]Department of Nutritional Science, School of Life Course Sciences, Faculty of Life Sciences & Medicine, King's College London, Franklin Wilkins Building, London SE1 9NH, UK, Christelle Elia research assistant, Seeromanie Harding professor in social epidemiology

[3] Institute of Psychiatry, Federal University of Rio de Janeiro (UFRJ), Rio de Janeiro, Brazil CEP 22290-140 Clayton Peixoto PhD candidate

[4]Institute of Psychiatry, Psychology and Neuroscience, Kings College London, Denmark Hill Campus, London SE5 9RJ, UK, Paola Dazzan professor of neurobiology of psychosis

[5]Cardiovascular Medicine & Social Epidemiology group, Division of Diabetes & Nutritional Sciences, Kings College London, Franklin Wilkins Building, London SE1 9NH, UK, J Kennedy Cruickshank professor of cardiovascular medicine & diabetes

Word count: 3098

Corresponding author:
Antonio Jose Grande
Av. Dom Antônio Barbosa, 4155 - Vila Santo Amaro, Campo Grande
Mato Grosso do Sul, Brazil. CEP 79115-898
Phone Number +55 67 34115326
email: grandeto@gmail.com

Transparency statement The corresponding author attests that all listed authors meet authorship criteria and that no others meeting the criteria have been omitted.

For peer review only

Title: Mental health interventions for suicide prevention among Indigenous adolescents: a systematic review protocol

Abstract

Introduction: There are more than 370 million Indigenous people from 5,000 cultures living in 90 countries worldwide. Although they make up 5% of the global population, they account for 15% of the extreme poor. Youth suicide is the second leading cause of mortality among 15-29year olds and disproportionately affects Indigenous youth. This research protocol pertains to a systematic review of studies that use a comparator/control group to evaluate the effectiveness of suicide interventions targeting Indigenous adolescents (aged 10-19 years).

Methods and analysis: We will conduct a systematic search on MEDLINE, EMBASE, CINAHL, LILACS, PsycINFO from inception to September 2019 to identify articles that compare mental health interventions for suicide prevention among Indigenous adolescents. Two reviewers will independently determine the eligibility of each study. Studies will be assessed for methodological quality using the risk of bias tool to assess non-randomized studies of interventions. We will conduct a meta-analysis if possible and use established statistical methods to identify and control for heterogeneity where appropriate.

Ethics and dissemination: This systematic review will use published data and does not require ethical permission is not required. However, this review is in preparation of a feasibility study that will examine how best to support the physical and mental health of indigenous adolescents in Brazil. Ethics approval for the feasibility study was obtained from National Research Ethics Commission. Findings will be disseminated through a peer-reviewed publication and will be made available to key decision-makers with authority for Indigenous health and other relevant stakeholders.

Registration details: The study protocol is registered at International prospective register of systematic reviews (PROSPERO) ID number 141754.

Keywords: Indigenous, adolescents, suicide prevention, mental health, interventions

ARTICLE SUMMARY

Strength and limitations of this study

- The systematic review will fill a gap in the evidence-base by providing a comprehensive assessment of the existing literature that evaluate the effectiveness of mental health interventions for suicide prevention among Indigenous adolescents.
- Rigorous methods of review with be followed with at least two independent study authors to conduct for screening, data extraction and critical appraisal.
- The findings will inform policy actors and practitioners about feasible interventions to prevent suicide among Indigenous adolescents.

For peer review only

- If a paucity of studies is reported then this would locate a research gap that needs to be urgently addressed.

INTRODUCTION

There are more the 370 million Indigenous people from 5,000 cultures living in 90 countries worldwide. Although they make up 5% of the global population, they account for 15% of the extreme poor [1]. Anderson et al (2016) systematic analysis of several social and health indicators for 28 indigenous populations in 23 countries showed poorer outcomes for Indigenous peoples than for non-Indigenous populations [2]. A gap in life expectancy at birth (that is lower in Indigenous than non-Indigenous populations in the same country) of more than 5 years was recorded for Indigenous populations in Australia, Cameroon, Canada (First Nations and Inuit), Greenland, Kenya, New Zealand, and Panama. Infant mortality rates for indigenous infants were more than twice those observed for non-indigenous or national populations in Brazil, Colombia, Greenland, Peru, Russia, and Venezuela. Poverty, poor education levels, employment status, and access to health services are all important contributors to the health disparities. Despite representing a rich diversity of cultures, they continue to be among the world's most disadvantaged groups, regardless of whether they live-in high-income countries (e.g. the Inuit in Canada) or lower-middle income countries (e.g. Baka in Cameroon). The legacy of colonization and of policies of forced assimilation continue to be a cause of intergenerational trauma, manifested through feelings of marginality, depression, anxiety and confusion, which places indigenous peoples at increased risk of suicide[3].

Youth suicide is the second leading cause of mortality among 15-29 years old[4] and disproportionately affects Indigenous youth[5,6,7]. Indigenous children (5–17 years old) in Australia die from suicide at five times the rate of their non-Indigenous peers (10.1 per 100 000 vs 2 per 100 000 in 2013–17). Similarly, in New Zealand, the suicide rate in Maori youth aged 15–24 years is 40.7 per 100 000 (vs 15.6 per 100 000 in non-Maori youth), and in Inuit youth in Canada the figure is 11 times the non-Indigenous average[8]. Most interpretations of this gap highlight the persistent social and economic disadvantage experienced by Indigenous youth relative to non-Indigenous youth[9]. The epidemic of youth suicide is relatively recent in some cultures, increased over time, more so in the latter half of the 20th century, with men accounting for the majority of suicides, and the 15-24 years old group having the highest suicide rates of any age group [10,11]. Furthermore, suicide among Indigenous young people may be unreported due to misclassification. Risk factors include mental health disorders, stressful life events, substance abuse, and poor physical health all of which occur at disproportionately higher rates in Indigenous populations[12,13]. Suicide in youth is also known to occur in clusters, is one of the strongest risk factors for death by suicide, and relates to depression, conduct disorders and substance and alcohol abuse[6]. Protective factors include high support (social and familiar), and cultural connectedness and personality factors such as high self-esteem and internal locus of control and increasing age[6].

For peer review only - http://bmjopen.bmj.com/site/about/guidelines.xhtml

Over the last 20 years, Indigenous people's rights have been increasingly recognised through international organisations such as the United Nations Permanent Forum on Indigenous Issues, which has a permanent forum for youth[2]. The 2030 Agenda for Sustainable Development refers to Indigenous people 6 times: 3 times in the political declaration, twice in the target under Goal 2 on Zero Hunger (target 2.3) and Goal 4 on education (target 4.5). Many of the Sustainable Development Goals (SDGs), however, are relevant for Indigenous peoples, particularly the focus on reducing inequalities and reducing mortality from non-communicable diseases (including suicide) by 33% by 2030. Given the vulnerability of Indigenous communities, implementation of SDGs could use the opportunity to develop initiatives for better outcomes among Indigenous communities. With such high rates of suicide among Indigenous youth [8], culturally tailored suicide interventions are urgently needed. Many indigenous populations hold a holistic view of health and well-being and interventions need to align with these perspectives and also engage with the economic, socio-environmental and historical issues that contribute to youth suicide in Indigenous cultures. There have been two reviews of suicide prevention programmes [9,10]. Both captured studies published up to 2012. Clifford, Doran and Tsey (2013) [10] reported on two Australian programs and seven American programs. The programmes targeted all ages, and there was a general lack of rigorous evaluation as only one study included in the review evaluated outcomes using a comparator group. Harlow and Clough (2014) [9] reported on nine evaluations of suicide prevention interventions with youths; five targeting Native Americans; three targeting Aboriginal Australians; and one targeting First Nation Canadians. As with the previous review, poor evaluation designs were noted. In recognition of a general lack of methodologically rigorous study designs across geographically and culturally diverse Indigenous populations and the need for an updated review, we will use a broad eligibility criterion to maximise the possibility of capturing any study that attempted to evaluate prevention programmes using a comparator group among adolescents.
This article presents the protocol for a systematic review of studies which use a comparator/control group to evaluate the effectiveness of suicide interventions targeting Indigenous adolescents (aged 10-19 years).

OBJECTIVES

To synthesize the scientific evidence on suicide prevention programmes targeting Indigenous youth

METHODS AND ANALYSIS

This protocol adheres to the Preferred Reporting Items for Systematic reviews and Meta-Analyses Protocols (PRISMA-Equity) guidelines[14]. The study protocol is registered at International prospective register of systematic reviews (PROSPERO) ID number 141754.

Types of studies

We will include any randomised or non-randomised studies, which has a control or comparative group.

Types of participants

For peer review only - http://bmjopen.bmj.com/site/about/guidelines.xhtml

The participants will be adolescents aged 10-19 years who self-identify as Indigenous peoples and are accepted as such by their community[2]. We are guided by the policy definition developed by the International Labour Organisation in 1989. It characterises Indigenous peoples as: tribal peoples in independent countries whose social, cultural, and economic conditions distinguish them from other sections of the national community and whose status is regulated wholly or partly by their own customs or traditions or by special laws or regulations; and peoples in independent countries who are regarded as indigenous because of their descent from the populations who inhabited the country, or a geographical region to which the country belongs, at the time of conquest or colonisation or the establishment of present state boundaries and who, irrespective of their legal status, retain some or all of their own social, economic, cultural, and political institutions [15,16].

We plan to investigate mental health problems as defined by the DSM-V.

Types of interventions

We will include in person or e-health interventions and which have targeted young Indigenous people anywhere in the world. We will consider a wide range of delivery channels (e.g., in person, online, phone), different practitioners (healthcare practitioners, teachers, lay health care providers) and sectors (i.e., health, primary, secondary and tertiary care, education, guardianship councils).

Types of outcome measures

Primary outcomes

- Self-injury acts
- Suicidal ideation
- Suicide attempts
- Death by suicide

Secondary outcomes

- Wellbeing / Quality of life

- Social Functioning including Educational Outcomes

Search methods for identification of studies

Electronic searches

We will search the Cochrane Central Register of Controlled Trials (CENTRAL, March 2020, MEDLINE (1966 to March 2020), EMBASE (1974 to March 2020), CINAHL (1981 to March 2020), LILACS (Latin American and Caribbean Health Sciences) (1982 to March 2020), PsycINFO (1887 to March 2020). We will use the search terms: (Indigenous or Indigenous or native* or Native* or Māori or Maori or Aborigin* or aboriginal or “Torres Strait Island*” or “torres strait island*” or “first nation*” or “first people*” or Inuit or Metis or Métis or ethnic* or “population groups”) AND (intervention* or program* or treatment* or treat* or therap* or service* or prevent* or diversion* or

For peer review only

initiative*) AND (wellbeing or “well being” or mental or depress* or anx* or suicide* or trauma* or alcohol* or drinking or cannabis or cocaine or methamphet* or amphet* or substance* or addict* or heal* or empower* or grief or loss* or stress* or psychosis or psychoses or psychotic or resilien* or recovery or “mental health” OR schizophrenia or mania or mood or internalizing or externalizing or affective or behavioural or drugs or “crack cocaine” or addiction or “mental illness” or happiness or emotion* or psych* or psychology) adapted to every other database. We will not use any language restrictions. If articles are not in English, Italian, Lebanese or Portuguese (native languages of the authors), we will use academic networks (e.g. Cochrane) to translate the critical parts (methods and results) to enable screening of abstracts and, if included this will be done for the full paper

Searching other resources

Experts will be identified via professional organizations (e.g. Royal College of Psychiatrists), academic networks and research societies (Social Science and Medicine). Also, Brazilian medical association and Researchers at Federal and State universities of Brazil who study indigenous health. We will look for published and unpublished studies that may be eligible for inclusion.

Inclusion criteria

We will include non-randomised and randomised studies. Electronic database search for theses and dissertations. We will also include studies that used data collection techniques (individual interviews, focus groups and observation) or data analysis processes characteristic of the qualitative method (for example: thematic analysis, content analysis).

Our primary aim focus is on any intervention that prevent suicide, which may also include studies that used positive psychology and promoted resilience-related competencies, where these were implemented for suicide prevention. These competencies may include the use of coping skills, that may ultimately mitigate the emergence of acute states of distress and mental health problems to eventually prevent suicidal behaviour. Improved social-emotional competence can be expected to afford some degree of protection against the development of suicidal ideations and behaviours. Hence, identifying and understanding aspects of interventions (e.g. use of problem solving for conflict resolution, community/cultural assets,) that promote resilience is an important step in preventing suicide [17-18].

Additionally, we aim to capture a broad range of interventions that include either a component, or solely targeting suicide prevention in adolescents in the education and community sectors. These may include suicide-specific education programmes, combined suicide-specific education and life skills training programmes, individual-level psychotherapeutic interventions, gatekeeper training, peer/community-help, peer gatekeeper training, and curriculum-based interventions. These have now been listed among the inclusion criteria and the title has been refined accordingly.

We will also consider the following inclusion criteria:

For peer review only - http://bmjopen.bmj.com/site/about/guidelines.xhtml

• Self-identification as Indigenous peoples by individuals and acceptance as such by their community According to International Labour Organization (ILO 1989)[2].
• Historical continuity and land occupation before invasion and colonisation
• Strong links to territories (land and water) and related natural resources
• Distinct social, economic, or political systems
• Distinct language, culture, religion, ceremonies, and beliefs
• Tendency to form non-dominant groups of society
• Resolution to maintain and reproduce ancestral environments and systems as distinct peoples and communities
• Tendency to manage their own affairs separate from centralised state authorities

Exclusion criteria

If the population included in study is not Indigenous or there is no distinction between Indigenous and non-Indigenous populations.

If youth are not included in study or are only briefly mentioned or adult population.

Data collection and analysis

Two review authors (AJG, CP) will independently assess all studies identified from the database searches by screening titles and abstracts using EndNoteX8 software. A third review author (SH) resolved any disagreements, and reasons for including and excluding trials were recorded. Next, AJG and CP will independently assess the full-text reports for inclusion against the selection criteria.

After both authors have discussed the results of the selection process and have made a consensus decision on which articles to be included/excluded, the data from each of the articles meeting the eligibility criteria will be extracted.

Qualitative data in the included studies will be reported using a narrative synthesis. Themes reported in the individual studies will be described in a table, including any reported for barriers and facilitators. All the reviewers will initially generate the analytical themes independently and then collectively as a group so as to minimize bias.

Data extraction and management

Two review authors (AJG, CP) will independently extract data from the included studies using a standard data extraction form.

A standardised, pre-piloted form will be used to extract data from the included studies for assessment of study quality and evidence synthesis. Missing data will be requested from study authors. The draft format will include:

Study details: aim, study design including whether a feasibility study was conducted in collaboration with the community to co-develop the design, design details (including evaluation), country in which study was conducted, details on location of intervention delivery (i.e., city or community), target condition / risk factor (i.e., subthreshold symptoms, experience of child maltreatment).

For peer review only - http://bmjopen.bmj.com/site/about/guidelines.xhtml

Participants: sample size (intervention and control groups at baseline and follow-up), sociodemographic characteristics (e.g., age, gender, ethnicity, socioeconomic status), attrition from the study.

Intervention details: description of intervention including frequency and duration of treatments /sessions, mode of delivery (face to face, internet), format (one to one or group), cultural appropriate content, cost of intervention

Delivery of the intervention: setting in which intervention was delivered (school, home, healthcare practice), who delivered the intervention (i.e., medical doctor, nurse, psychologist, teacher, lay health worker, peer promotion etc) and whether it was delivered by one practitioner or a team of individuals or online, fidelity of implementers to protocol, culturally appropriate modes of delivery and whether there was intersectoral collaboration (i.e., between health and education or guardianship councils).

The RE -AIM framework will used to enhance the assessment of program elements that can improve the sustainable adoption and implementation of effective, generalised/localised, evidence-based interventions [19]. RE-AIM targets Reach of the target population; Effectiveness or efficacy of the intervention (impact of an intervention on important outcomes, including potential negative effects, quality of life, and economic outcomes); adoption by target staff, settings, or institutions; Implementation consistency, costs and adaptions made during delivery; and Maintenance of intervention effects in individuals and settings over time

Assessment of risk of bias in included studies

Cognisant of the well documented limitations of the use of 'western' methods in an indigenous context, our critical appraisal will include identifying culturally appropriate methodologies such as Storytelling and Community-Based Participatory Research, with the inclusion of Indigenous Peoples in the research process in a way that is respectful and reciprocal. We will include comparator groups as well as randomised study designs in recognition that the former may be more appropriate for the indigenous context [20,21].

Two review authors (AJG, CP) will independently assess the risk of bias of the included studies using the Risk Of Bias In Non-randomized Studies - of Interventions. (ROBINS I) for non-randomized studies and Risk of Bias tool 2.0 for randomized studies.

Measures of treatment effect

Types of measurements of treatment effect that may be used**:**

1. Dichotomous data: we will use risk ratio for likely binary outcomes, prevalence ratio for some outcomes.

2. Continuous data: we will combine the results using the mean difference for measures using the same scale or the standardised mean difference where different scales have been used to evaluate the same outcome

Unit of analysis issues

For peer review only - http://bmjopen.bmj.com/site/about/guidelines.xhtml

We will consider the individual as the unit of analysis. We will separate the non-randomised from randomised studies if we have enough studies for meta-analysis.

Dealing with missing data

We will send two emails (one initial, one reminder) to the corresponding author to ask for any missing data or incompletely reported study details. We will check for consistency between studies and analyse each outcome.

Assessment of heterogeneity

We will assess the inconsistencies between studies using the I^2 statistic which gives the percentage of total variation across studies that is due to heterogeneity rather than chance. We will consider heterogeneity substantial if I2 is over 50%. See below for subgroup analysis.

Assessment of reporting biases

If mismatches are identified between study protocols and reports, we will contact the trial authors to clarify the information. We plan to explore the impact of including such studies by conducting a sensitivity analysis. We will perform a funnel plot asymmetry test if 10 or more trials are included.

Data synthesis

We will present the data separately for randomized and non-randomized studies. We will meta-analyse trials if the combination of data for the outcomes is possible. We will use a random-effects model, independently of the heterogeneity identified. Forest plot graphics produced by RevMan 5.3 will illustrate meta-analyses. If the combination of data is not possible, we will present a narrative analysis of individual studies. We will create a 'Summary of findings' table using the outcomes proposed in this protocol and we will present the quality of the body of evidence considering the five GRADE assumptions (study limitations, consistency of effect, imprecision, indirectness and publication bias) contributing to the meta-analyses for the pre-specified outcomes.

Subgroup analysis and investigation of heterogeneity

We plan to explore the subsets or subgroups of countries/regions, for primary, secondary or tertiary care, type of mental health condition, gender, age, urban/rural area.

Sensitivity analysis

We will pool included studies to verify whether the impact of risk of bias affects the overall effect. We will explore which studies increased heterogeneity.

Ethics and dissemination

This study is part of wider feasibility study that aims to examine how best to support the physical and mental health of indigenous adolescents in Brazil. Ethics approval for the feasibility study was obtained from National Research Ethics Commission (CONEP), protocol CAAE: 89604318.0000.8030, analysis #3.100.358, from December of 2018.

For peer review only - http://bmjopen.bmj.com/site/about/guidelines.xhtml

This systematic review will use published data; therefore, ethical permission is not required. Findings will be published in a peer-reviewed scientific journal. Our results will also be presented at national and international conferences and will be made available to key decision-makers with authority for Indigenous health.

Quality of the evidence

Two review authors (AJG, CP) will independently rate the quality of the outcomes. We will use GRADE to rank the quality of the evidence using the GRADEprofiler Guideline Development Tool software[22] and the guidelines provided in Chapter 11 of the Cochrane Handbook for Systematic Reviews of Interventions[23].

Patient and Public Involvement

The topic to be covered by the review comes from our empirical experience with the Indigenous communities in Brazil and from consultations with researchers in indigenous health, and practitioners and policy actors responsible for indigenous health. The Indigenous people were not directly involved in the writing of this protocol. The results will be disseminated to indigenous communities, practitioners, and policy actors to aid the planning and provision of services. The authors are from Brazil and UK and do not self-identify as Indigenous.

CONCLUSION

This review will present the best-available evidence for decision making on suicide prevention programmes targeting Indigenous youth in the literature. It will provide in one single document all the primary research on the topic with the quality assessment for each single paper and will rank the quality of evidence produced.

References

1. World Bank (2019) 'Understanding Poverty. Indigenous People', available online https://www.worldbank.org/en/topic/IndigenousPeoples (accessed July 17th, 2019).
2. Anderson I, Robson B, Connolly M, Al-Yaman F, Bjertness E, King A, Tynan M, Madden R, Bang A, Coimbra CE Jr, Pesantes MA, Amigo H, Andronov S, Armien B, Obando DA, Axelsson P, Bhatti ZS, Bhutta ZA, Bjerregaard P, Bjertness MB, Briceno-Leon R, Broderstad AR, Bustos P, Chongsuvivatwong V, Chu J, Deji, Gouda J, Harikumar R, Htay TT, Htet AS, Izugbara C, Kamaka M, King M, Kodavanti MR, Lara M, Laxmaiah A, Lema C, Taborda AM, Liabsuetrakul T, Lobanov A, Melhus M, Meshram I, Miranda JJ, Mu TT, Nagalla B, Nimmathota A, Popov AI, Poveda AM, Ram F, Reich H, Santos RV, Sein AA, Shekhar C, Sherpa LY, Skold P, Tano S, Tanywe A, Ugwu C, Ugwu F, Vapattanawong P, Wan X, Welch JR, Yang G, Yang Z, Yap L. Indigenous and tribal peoples' health (The Lancet-Lowitja Institute Global Collaboration): a population study. Lancet. 2016 Jul 9;388(10040):131-57.
3. Pollock NJ, Apok C, Concepcion T, Delgado RA Jr, Rasmus S, Chatwood S, Collins PY. Global goals and suicide prevention in the Circumpolar North. Indian J Psychiatry. 2020 Jan-Feb;62(1):7-14.

For peer review only - http://bmjopen.bmj.com/site/about/guidelines.xhtml

4. Lawson-Te Aho K, Liu JH. Indigenous suicide and colonization: The legacy of violence and the necessity of self-determination. International Journal of Conflict and Violence (IJCV). 2010 May 7;4(1):124-33.
5. Patton GC, Coffey C, Sawyer SM, Viner RM, Haller DM, Bose K, Vos T, Ferguson J, Mathers CD. Global patterns of mortality in young people: a systematic analysis of population health data. The lancet. 2009 Sep 12;374(9693):881-92.
6. Harder HG, Rash J, Holyk T, Jovel E, Harder K. Indigenous youth suicide: a systematic review of the literature. Pimatisiwin: A Journal of Aboriginal and Indigenous Community Health. 2012;10(1):125-42.
7. Hunter E, Harvey D. Indigenous suicide in Australia, New Zealand, Canada and the United States. Emergency Medicine. 2002 Mar;14(1):14-23.
8. Pollock NJ, Naicker K, Loro A, Mulay S, Colman I. Global incidence of suicide among Indigenous peoples: a systematic review. BMC medicine. 2018 Dec;16(1):145.
9. Harlow AF, Clough A. A systematic review of evaluated suicide prevention programs targeting Indigenous youth. Crisis. 2014.
10. Clifford AC, Doran CM, Tsey K. A systematic review of suicide prevention interventions targeting Indigenous peoples in Australia, United States, Canada and New Zealand. BMC public health. 2013 Dec;13(1):463.
11. Gracey M, King M. Indigenous health part 1: determinants and disease patterns. The Lancet. 2009 Jul 4;374(9683):65-75.
12. King M, Smith A, Gracey M. Indigenous health part 2: the underlying causes of the health gap. The lancet. 2009 Jul 4;374(9683):76-85.
13. United Nations Division for Sustainable Development (2018b) 'Indigenous Peoples and the Post-2015 Development Agenda', available online at https://www.un.org/development/desa/IndigenousPeoples/focus-areas/post-2015agenda.html (accessed May 8, 2019)
14. Welch V, Petticrew M, Tugwell P, Moher D, O'Neill J, Waters E, et al. (2012) PRISMA-Equity 2012 Extension: Reporting Guidelines for Systematic Reviews with a Focus on Health Equity. PLoS Med 9(10): e1001333.
15. UN Department of Economic and Social Aff airs. The concept of indigenous peoples. New York: United Nations, 2004.
16. UN Permanent Forum on Indigenous Issues. Who are indigenous peoples? http://www.un.org/esa/socdev/unpfi i/ documents/5session_factsheet1.pdf (accessed Feb 4, 2020).
17. Dickson JM, Cruise K, McCall CA, Taylor PJ. A Systematic Review of the Antecedents and Prevalence of Suicide, Self-Harm and Suicide Ideation in Australian Aboriginal and Torres Strait Islander Youth. Int J Environ Res Public Health. 2019 Aug 29;16(17):3154. doi: 10.3390/ijerph16173154. PMID: 31470580; PMCID: PMC6747538.
18. Bjorkenstam C, Kosidou K, Bjorkenstam E. Childhood adversity and risk of suicide: cohort study of 548 721 adolescents and young adults in Sweden. Bmj-Brit Med J. 2017;357.
19. Gaglio B, Shoup JA, Glasgow RE. The RE-AIM framework: a systematic review of use over time. Am J Public Health. 2013 Jun;103(6):e38-46.Eder, D.J. (2007). Bringing Navajo storytelling practices into schools: the importance of maintaining cultural integrity. Anthropol. Educ. Q., 38, 278-296.
20. Bachmann S. Epidemiology of Suicide and the Psychiatric Perspective. Int J Env Res Pub He. 2018;15(7).

For peer review only - http://bmjopen.bmj.com/site/about/guidelines.xhtml

21. Tobias, J. K., Richmond, C. A., & Luginaah, I. (2013). Community- based participatory research (CBPR) with indigenous communities: Producing respectful and reciprocal research. Journal of Empirical Research on Human Research Ethics, 8(2), 129–140.
22. GRADEpro Guideline Development Tool [Software] [Computer program]. McMaster University (developed by Evidence Prime, Inc.), Available from www.gradepro.org, 2015.
23. Schünemann HJ, Oxman AD, Higgins JPT, Vist GE, Glasziou P, Akl E, et al, on behalf of the Cochrane GRADEing Methods Group and the Cochrane Statistical Methods Group. Chapter 11: Completing 'Summary of findings' tables and grading the confidence in or quality of the evidence. In: Higgins JPT, Churchill R, Chandler J, Cumpston MS, editor(s). Cochrane Handbook for Systematic Reviews of Interventions version 5.2.0 (updated June 2017). Cochrane, 2017 (available from www.training.cochrane.org/handbook).

Funding statement This work was supported by the Medical Research Council (MRC) grant number MR/R022739/1. Fundação de Apoio ao Desenvolvimento do Ensino, Ciência e Tecnologia do Estado de Mato Grosso do Sul, grant number 71/700070/2018

Authors' contributions AJG and CE wrote the protocol and approval the final version of the protocol; CP, PJ, PD, AV, JC, SH co-wrote the protocol, critical reviewed and approved the final version.

Competing interests: The authors declare no competing interests

For peer review only

Reporting checklist for protocol of a systematic review.

Based on the PRISMA-P guidelines.

Instructions to authors

Complete this checklist by entering the page numbers from your manuscript where readers will find each of the items listed below.

Your article may not currently address all the items on the checklist. Please modify your text to include the missing information. If you are certain that an item does not apply, please write "n/a" and provide a short explanation.

Upload your completed checklist as an extra file when you submit to a journal.

In your methods section, say that you used the PRISMA-Preporting guidelines, and cite them as:

Moher D, Shamseer L, Clarke M, Ghersi D, Liberati A, Petticrew M, Shekelle P, Stewart LA. Preferred Reporting Items for Systematic Review and Meta-Analysis Protocols (PRISMA-P) 2015 statement. Syst Rev. 2015;4(1):1.

		Reporting Item	Page Number
**Title**			
Identification	#1a	Identify the report as a protocol of a systematic review	1,2
Update	#1b	If the protocol is for an update of a previous systematic review, identify as such	N/A
**Registration**			
	#2	If registered, provide the name of the registry (such as PROSPERO) and registration number	2
**Authors**			
Contact	#3a	Provide name, institutional affiliation, e-mail address of all protocol authors; provide physical mailing address of corresponding author	1
Contribution	#3b	Describe contributions of protocol authors and identify the guarantor of the review	1,9

For peer review only

**Amendments**			
	#4	If the protocol represents an amendment of a previously completed or published protocol, identify as such and list changes; otherwise, state plan for documenting important protocol amendments	
**Support**			
Sources	#5a	Indicate sources of financial or other support for the review	9
Sponsor	#5b	Provide name for the review funder and / or sponsor	9
Role of sponsor or funder	#5c	Describe roles of funder(s), sponsor(s), and / or institution(s), if any, in developing the protocol	9
**Introduction**			
Rationale	#6	Describe the rationale for the review in the context of what is already known	3,4
Objectives	#7	Provide an explicit statement of the question(s) the review will address with reference to participants, interventions, comparators, and outcomes (PICO)	4
**Methods**			
Eligibility criteria	#8	Specify the study characteristics (such as PICO, study design, setting, time frame) and report characteristics (such as years considered, language, publication status) to be used as criteria for eligibility for the review	4
Information sources	#9	Describe all intended information sources (such as electronic databases, contact with study authors, trial registers or other grey literature sources) with planned dates of coverage	5
Search strategy	#10	Present draft of search strategy to be used for at least one electronic database, including planned limits, such that it could be repeated	Appendix
Study records - data management	#11a	Describe the mechanism(s) that will be used to manage records and data throughout the review	6
Study records - selection process	#11b	State the process that will be used for selecting studies (such as two independent reviewers) through each phase of the review (that is, screening, eligibility and inclusion in meta-analysis)	6

For peer review only

Study records - data collection process	#11c	Describe planned method of extracting data from reports (such as piloting forms, done independently, in duplicate), any processes for obtaining and confirming data from investigators	6
Data items	#12	List and define all variables for which data will be sought (such as PICO items, funding sources), any pre-planned data assumptions and simplifications	6
Outcomes and prioritization	#13	List and define all outcomes for which data will be sought, including prioritization of main and additional outcomes, with rationale	4,5
Risk of bias in individual studies	#14	Describe anticipated methods for assessing risk of bias of individual studies, including whether this will be done at the outcome or study level, or both; state how this information will be used in data synthesis	7
Data synthesis	#15a	Describe criteria under which study data will be quantitatively synthesised	7
Data synthesis	#15b	If data are appropriate for quantitative synthesis, describe planned summary measures, methods of handling data and methods of combining data from studies, including any planned exploration of consistency (such as I2, Kendall's τ)	
Data synthesis	#15c	Describe any proposed additional analyses (such as sensitivity or subgroup analyses, meta-regression)	
Data synthesis	#15d	If quantitative synthesis is not appropriate, describe the type of summary planned	7
Meta-bias(es)	#16	Specify any planned assessment of meta-bias(es) (such as publication bias across studies, selective reporting within studies)	
Confidence in cumulative evidence	#17	Describe how the strength of the body of evidence will be assessed (such as GRADE)	8

None The PRISMA-P checklist is distributed under the terms of the Creative Commons Attribution License CC-BY 4.0. This checklist can be completed online using https://www.goodreports.org/, a tool made by the EQUATOR Network in collaboration with Penelope.ai

For peer review only

BMJ Open

Mental health interventions for suicide prevention among indigenous adolescents: a systematic review protocol

Journal:	*BMJ Open*
Manuscript ID	bmjopen-2019-034055.R2
Article Type:	Protocol
Date Submitted by the Author:	10-Mar-2020
Complete List of Authors:	Grande, Antonio José ; Mato Grosso State University Medical Course<br>Elia, Christelle; King's College London Faculty of Life Sciences and Medicine<br>Peixoto, Clayton; Federal University of Rio de Janeiro<br>Jardim, Paulo de Tarso Coelho; State University of Mato Grosso do Sul<br>Dazzan, Paola; King's College London Faculty of Life Sciences and Medicine<br>Veras, Andre Barciela; State University of Mato Grosso do Sul<br>Cruickshank, John; King's College London Faculty of Life Sciences and Medicine<br>Harding, Seeromanie; King's College London Faculty of Life Sciences and Medicine
**Primary Subject Heading**:	Mental health
Secondary Subject Heading:	Evidence based practice, Epidemiology, Mental health, Global health, Health policy
Keywords:	Suicide & self-harm < PSYCHIATRY, Adolescent, Indigenous, MENTAL HEALTH, Child & adolescent psychiatry < PSYCHIATRY, PUBLIC HEALTH

SCHOLARONE™
Manuscripts

I, the Submitting Author has the right to grant and does grant on behalf of all authors of the Work (as defined in the below author licence), an exclusive licence and/or a non-exclusive licence for contributions from authors who are: i) UK Crown employees; ii) where BMJ has agreed a CC-BY licence shall apply, and/or iii) in accordance with the terms applicable for US Federal Government officers or employees acting as part of their official duties; on a worldwide, perpetual, irrevocable, royalty-free basis to BMJ Publishing Group Ltd ("BMJ") its licensees and where the relevant Journal is co-owned by BMJ to the co-owners of the Journal, to publish the Work in this journal and any other BMJ products and to exploit all rights, as set out in our licence.

The Submitting Author accepts and understands that any supply made under these terms is made by BMJ to the Submitting Author unless you are acting as an employee on behalf of your employer or a postgraduate student of an affiliated institution which is paying any applicable article publishing charge ("APC") for Open Access articles. Where the Submitting Author wishes to make the Work available on an Open Access basis (and intends to pay the relevant APC), the terms of reuse of such Open Access shall be governed by a Creative Commons licence – details of these licences and which Creative Commons licence will apply to this Work are set out in our licence referred to above.

Other than as permitted in any relevant BMJ Author's Self Archiving Policies, I confirm this Work has not been accepted for publication elsewhere, is not being considered for publication elsewhere and does not duplicate material already published. I confirm all authors consent to publication of this Work and authorise the granting of this licence.

For peer review only

Title page

Title: Mental health interventions for suicide prevention among Indigenous adolescents: a systematic review protocol

Authors:

Antonio José Grande[1] 0000-0001-7182-075, Christelle Elia[2], Clayton Peixoto[3], Paulo de Tarso Coelho Jardim[1], Paola Dazzan[4], Andre Barciela Veras[1], John Kennedy Cruickshank[5], Seeromanie Harding[2]

[1]Medical Course, State University of Mato Grosso do Sul, Campo Grande, Mato Grosso do Sul, Brazil,CEP 79115-898, Antonio Jose Grande adjunct professor, Paolo de Tarso Coelho adjunct professor

[2]Department of Nutritional Science, School of Life Course Sciences, Faculty of Life Sciences & Medicine, King's College London, Franklin Wilkins Building, London SE1 9NH, UK, Christelle Elia research assistant, Seeromanie Harding professor in social epidemiology

[3] Institute of Psychiatry, Federal University of Rio de Janeiro (UFRJ), Rio de Janeiro, Brazil CEP 22290-140 Clayton Peixoto PhD candidate

[4]Institute of Psychiatry, Psychology and Neuroscience, Kings College London, Denmark Hill Campus, London SE5 9RJ, UK, Paola Dazzan professor of neurobiology of psychosis

[5]Cardiovascular Medicine & Social Epidemiology group, Division of Diabetes & Nutritional Sciences, Kings College London, Franklin Wilkins Building, London SE1 9NH, UK, J Kennedy Cruickshank professor of cardiovascular medicine & diabetes

Word count: 3098

Corresponding author:
Antonio Jose Grande
Av. Dom Antônio Barbosa, 4155 - Vila Santo Amaro, Campo Grande
Mato Grosso do Sul, Brazil. CEP 79115-898
Phone Number +55 67 34115326
email: grandeto@gmail.com

Transparency statement: The corresponding author attests that all listed authors meet authorship criteria and that no others meeting the criteria have been omitted.

For peer review only

Title: Mental health interventions for suicide prevention among Indigenous adolescents: a systematic review protocol

Abstract

Introduction: There are more than 370 million Indigenous people from 5,000 cultures living in 90 countries worldwide. Although they make up 5% of the global population, they account for 15% of the extreme poor. Youth suicide is the second leading cause of mortality among 15-29year olds and disproportionately affects Indigenous youth. This research protocol pertains to a systematic review of studies that use a comparator/control group to evaluate the effectiveness of suicide interventions targeting Indigenous adolescents (aged 10-19 years).

Methods and analysis: We will conduct a systematic search on MEDLINE, EMBASE, CINAHL, LILACS, PsycINFO from inception to September 2019 to identify articles that compare mental health interventions for suicide prevention among Indigenous adolescents. Two reviewers will independently determine the eligibility of each study. Studies will be assessed for methodological quality using the risk of bias tool to assess non-randomised studies of interventions. We will conduct a meta-analysis if possible and use established statistical methods to identify and control for heterogeneity where appropriate.

Ethics and dissemination: This systematic review will use published data and does not require ethics approval. However, this review is in preparation of a feasibility study that will examine how best to support the physical and mental health of Indigenous adolescents in Brazil. Ethics approval for the feasibility study was obtained from National Research Ethics Commission. Findings will be disseminated through a peer-reviewed publication and will be made available to key decision-makers with authority for Indigenous health and other relevant stakeholders.

Registration details: The study protocol is registered at International prospective register of systematic reviews (PROSPERO) CRD42019141754.

Keywords: Indigenous, adolescents, suicide prevention, mental health, interventions

ARTICLE SUMMARY

Strength and limitations of this study

- The systematic review will fill a gap in the evidence-base by providing a comprehensive assessment of the existing literature that evaluate the effectiveness of mental health interventions for suicide prevention among Indigenous adolescents.
- Rigorous methods of review with be followed with at least two independent study authors to conduct for screening, data extraction and critical appraisal.

For peer review only - http://bmjopen.bmj.com/site/about/guidelines.xhtml

- The findings will inform policy actors and practitioners about feasible interventions to prevent suicide among Indigenous adolescents.
- If a paucity of studies is reported then this would locate a research gap that needs to be urgently addressed.

INTRODUCTION

There are more the 370 million Indigenous people from 5,000 cultures living in 90 countries worldwide. Although they make up 5% of the global population, they account for 15% of the extreme poor [1]. Anderson et al (2016) conducted a systematic analysis of several social and health indicators for 28 Indigenous populations in 23 countries and showed poorer outcomes for Indigenous peoples than for non-Indigenous populations [2]. A gap in life expectancy at birth (that is lower in Indigenous than non-Indigenous populations in the same country) of more than 5 years was recorded for Indigenous populations in Australia, Cameroon, Canada (First Nations and Inuit), Greenland, Kenya, New Zealand, and Panama. Infant mortality rates for Indigenous infants were more than twice those observed for non-Indigenous or national populations in Brazil, Colombia, Greenland, Peru, Russia, and Venezuela. Poverty, poor education levels, employment status, and access to health services are all important contributors to the health disparities. Despite representing a rich diversity of cultures, they continue to be among the world's most disadvantaged groups, regardless of whether they live-in high-income countries (e.g. the Inuit in Canada) or lower-middle income countries (e.g. Baka in Cameroon). The legacy of colonisation and of policies of forced assimilation continue to be a cause of intergenerational trauma, manifested through feelings of marginality, depression, anxiety and confusion, which places indigenous peoples at increased risk of suicide[3].

Youth suicide is the second leading cause of mortality among 15-29 years old[4] and disproportionately affects Indigenous youth[5,6,7]. Indigenous children (5–17 years old) in Australia die from suicide at five times the rate of their non-Indigenous peers (10.1 per 100 000 vs 2 per 100 000 in 2013–17). In New Zealand, the suicide rate in Maori youth aged 15–24 years is more than twice that non-Maori peers. (40.7 per 100 000 vs 15.6 per 100 000 in 2013–17). In Canada the rate among Inuit youth is 11 times that of the non-Indigenous youths [8]. Most interpretations of this gap highlight the persistent social and economic disadvantage experienced by Indigenous youth relative to non-Indigenous youth[9]. The epidemic of youth suicide is relatively recent in some cultures, with an increase in the latter half of the 20th century, with men accounting for the majority of suicides, and with the 15-24 years age group having the highest suicide rates of any age group [10,11]. Furthermore, suicide among Indigenous young people may be unreported due to misclassification. Risk factors include mental health disorders, stressful life events, substance abuse, and poor physical health all of which occur at disproportionately higher rates in Indigenous populations[12,13]. Suicide in youth is also known to occur in clusters, and suicidal behaviours (e.g. ideation, attempts) are strong risk factors for death by suicide[6]. Protective factors include high social support, cultural connectedness and personality factors such as high self-esteem and internal locus of control and increasing age[6].

For peer review only - http://bmjopen.bmj.com/site/about/guidelines.xhtml

Over the last 20 years, Indigenous people's rights have been increasingly recognised through international organisations such as the United Nations Permanent Forum on Indigenous Issues, which has a permanent forum for youth[2]. The 2030 Agenda for Sustainable Development refers to Indigenous people 6 times: 3 times in the political declaration, twice in the target under Goal 2 on Zero Hunger (target 2.3) and Goal 4 on education (target 4.5). Many of the Sustainable Development Goals (SDGs), however, are relevant for Indigenous peoples, particularly those with a focus on reducing inequalities and reducing mortality from non-communicable diseases (including suicide) by 33% by 2030. Given the vulnerability of Indigenous communities, implementation of the SDGs provide opportunities for policy actors to promote initiatives that improve outcomes among Indigenous communities. With such high rates of suicide among Indigenous youth [8], culturally relevant suicide interventions are urgently needed. Many Indigenous populations hold a holistic view of health and well-being and interventions need to align with these perspectives and engage with the economic, socio-environmental and historical issues that contribute to youth suicide in Indigenous cultures. There have been two reviews of suicide prevention programmes [9,10]. Both captured studies published up to 2012. Clifford, Doran and Tsey (2013) [10] reported on two Australian programs and seven American programs. The programmes targeted all ages, and there was a general lack of rigorous evaluation designs as only one study included in the review evaluated outcomes using a comparator group. Harlow and Clough (2014) [9] reported on nine evaluations of suicide prevention interventions with youths; five targeted Native Americans; three targeted Aboriginal Australians; and one targeted First Nation Canadians. As with the previous review, poor evaluation designs were noted. In recognition of a general lack of methodologically rigorous study designs across geographically and culturally diverse Indigenous populations and the need for an updated review, we will use a broad eligibility criterion to maximise the possibility of capturing any study that attempted to evaluate prevention programmes using a comparator group among adolescents.

This article presents the protocol for a systematic review of studies which used a comparator/control group to evaluate the effectiveness of suicide interventions targeting Indigenous adolescents (aged 10-19 years).

OBJECTIVE

To synthesise the scientific evidence on suicide prevention programmes targeting Indigenous youth. Our principal research question is: what interventions, including single or multi-component interventions, prevented suicides (or not) and why did they work (or not)?

METHODS AND ANALYSIS

This protocol adheres to the Preferred Reporting Items for Systematic reviews and Meta-Analyses Protocols (PRISMA-Equity) guidelines[14]. The study protocol is registered at International prospective register of systematic reviews (PROSPERO) CRD42019141754.

Types of studies

We will include any randomised or non-randomised studies, which has a control or comparative group.

For peer review only - http://bmjopen.bmj.com/site/about/guidelines.xhtml

Types of participants

The participants will be adolescents aged 10-19 years who self-identified as Indigenous peoples and are accepted as such by their community[2]. We are guided by the policy definition developed by the International Labour Organisation (ILO) in 1989. It characterises Indigenous peoples as: tribal peoples in independent countries whose social, cultural, and economic conditions distinguish them from other sections of the national community and whose status is regulated wholly or partly by their own customs or traditions or by special laws or regulations; and peoples in independent countries who are regarded as Indigenous because of their descent from the populations who inhabited the country, or a geographical region to which the country belongs, at the time of conquest or colonisation or the establishment of present state boundaries and who, irrespective of their legal status, retain some or all of their own social, economic, cultural, and political institutions [15,16].

Types of interventions

We will include in person or e-health interventions and which have targeted young Indigenous people anywhere in the world. We will consider a wide range of delivery channels (e.g., in person, online, phone), different practitioners (healthcare practitioners, teachers, lay health care providers) and sectors (i.e., health, primary, secondary and tertiary care, education, guardianship councils).

Types of outcome measures

Primary outcomes

- Self-injury acts
- Suicidal ideation
- Suicide attempts
- Death by suicide

Secondary outcomes

- Wellbeing / Quality of life

- Social Functioning including Educational Outcomes

Search methods for identification of studies

Electronic searches

We will search the Cochrane Central Register of Controlled Trials (CENTRAL, March 2020, MEDLINE (1966 to March 2020), EMBASE (1974 to March 2020), CINAHL (1981 to March 2020), LILACS (Latin American and Caribbean Health Sciences) (1982 to March 2020), PsycINFO (1887 to March 2020). We will use the search terms: (Indigenous or Indigenous or native* or Native* or Māori or Maori or Aborigin* or aboriginal or “Torres Strait Island*” or “torres strait island*” or “first nation*” or “first people*” or Inuit or Metis or Métis or ethnic* or “population groups”) AND (intervention* or program* or treatment* or treat* or therap* or service* or prevent* or diversion* or

For peer review only

initiative*) AND (wellbeing or “well being” or mental or depress* or anx* or suicide* or trauma* or alcohol* or drinking or cannabis or cocaine or methamphet* or amphet* or substance* or addict* or heal* or empower* or grief or loss* or stress* or psychosis or psychoses or psychotic or resilien* or recovery or “mental health” OR schizophrenia or mania or mood or internalizing or externalizing or affective or behavioural or drugs or “crack cocaine” or addiction or “mental illness” or happiness or emotion* or psych* or psychology) adapted to every other database. We will not use any language restrictions. If articles are not in English, Italian, Lebanese or Portuguese (native languages of the authors), we will use academic networks (e.g. Cochrane) to translate the critical parts (methods and results) to enable screening of abstracts and, if included this will be done for the full paper

Searching other resources

Experts will be identified via professional organizations (e.g. Royal College of Psychiatrists), academic networks and research societies (Social Science and Medicine), medical associations (.e.g, Brazilian Medical Association) and also via targeted researchers (e.g. those at Federal and State universities of Brazil who study Indigenous health). We will search for published and unpublished studies (e.g. theses available in electronic format) that may be eligible for inclusion.

Inclusion criteria

We will include non-randomised and randomised studies, regardless of whether they contained process evaluation although its inclusion in studies will aid the understanding of why an intervention worked or not.

Our primary aim focus is on any intervention that prevent suicide, which may also include studies that used positive psychology and promoted resilience-related competencies, where these were implemented for suicide prevention. These competencies may include the use of coping skills, that may ultimately mitigate the emergence of acute states of distress and mental health problems to eventually prevent suicidal behaviour. Improved social-emotional competence can be expected to afford some degree of protection against the development of suicidal ideations and behaviours. Hence, identifying and understanding aspects of interventions (e.g. use of problem solving for conflict resolution, community/cultural assets,) that promote resilience is an important step in preventing suicide [17-18].

Additionally, we aim to capture a broad range of interventions that include either a component, or solely targeting suicide prevention in adolescents in the education and community sectors. These may include suicide-specific education programmes, combined suicide-specific education and life skills training programmes, individual-level psychotherapeutic interventions, gatekeeper training, peer/community-help, peer gatekeeper training, and curriculum-based interventions.

Guided by the ILO definition, studies will be included if participants self-identified as indigenous and were accepted as such by their community According to International Labour Organization (ILO 1989)[2]; had historical continuity and land occupation before invasion and colonisation; had strong links to territories (land and water) and related natural resources; belonged to distinct social, economic, or political systems;

For peer review only - http://bmjopen.bmj.com/site/about/guidelines.xhtml

had distinct language, culture, religion, ceremonies, and beliefs; belonged to non-dominant groups of society with resolution to maintain and reproduce ancestral environments and systems as distinct peoples and communities, and with a tendency to manage their own affairs separate from centralised state authorities.

Exclusion criteria

If the population included in study is not Indigenous or there is no distinction between Indigenous and non-Indigenous populations.

If youth are not included in study or are only briefly mentioned or adult population.

Data collection and analysis

Two review authors (AJG, CE) will independently assess all studies identified from the database searches by screening titles and abstracts using EndNoteX8 software. A third review author (SH) will resolve any disagreements, and reasons for including and excluding trials were recorded. Next, AJG and CE will independently assess the full-text reports for inclusion against the selection criteria.

After both authors have discussed the results of the selection process and have made a consensus decision on which articles to be included/excluded, the data from each of the articles meeting the eligibility criteria will be extracted.

Qualitative data in the included studies will be reported using a narrative synthesis. Themes reported in the individual studies will be described in a table, including any reported for barriers and facilitators. All the reviewers will initially generate the analytical themes independently and then collectively as a group so as to minimise bias.

Data extraction and management

Two review authors (AJG, CE) will independently extract data from the included studies using a standard data extraction form.

A standardised, pre-piloted form will be used to extract data from the included studies for assessment of study quality and evidence synthesis. Missing data will be requested from study authors. The draft format will include:

Study details: aim, study design including whether a feasibility study was conducted in collaboration with the community to co-develop the design, design details, country in which study was conducted, details on location of intervention delivery (e.g.city or community), target condition / risk factor (e.g. subthreshold symptoms, experience of child maltreatment).

Participants: sample size (intervention and control groups at baseline and follow-up), sociodemographic characteristics (e.g., age, gender, ethnicity, socioeconomic status), attrition from the study.

For peer review only - http://bmjopen.bmj.com/site/about/guidelines.xhtml

Intervention details: description of intervention including frequency and duration of treatments /sessions, mode of delivery (face to face, internet), format (one to one or group), cultural appropriate content, cost of intervention

Delivery of the intervention: setting in which intervention was delivered (school, home, healthcare practice), who delivered the intervention (i.e., medical doctor, nurse, psychologist, teacher, lay health worker, peer promotion etc) and whether it was delivered by one practitioner or a team of individuals or online, fidelity of implementers to protocol, culturally appropriate modes of delivery and whether there was intersectoral collaboration (i.e., between health and education or guardianship councils).

The RE-AIM framework will be used to enhance the assessment of program elements that can improve the sustainable adoption and implementation of effective, generalised/localised, evidence-based interventions [19]. RE-AIM targets *Reach* of the target population; *Effectiveness* or efficacy of the intervention (impact of an intervention on important outcomes, including potential negative effects, quality of life, and economic outcomes); *Adoption* by target staff, settings, or institutions; *Implementation* consistency, costs and adaptions made during delivery; and *Maintenance* of intervention effects in individuals and settings over time

Assessment of risk of bias in included studies

Cognisant of the well documented limitations of the use of 'western' methods in an Indigenous context, our critical appraisal will include identifying culturally appropriate methodologies such as Storytelling and Community-Based Participatory Research, with the inclusion of Indigenous Peoples in the research process in a way that is respectful and reciprocal. We will include comparator groups as well as randomised study designs in recognition that the former may be more appropriate for the Indigenous context [20,21].

Two review authors (AJG, CE) will independently assess the risk of bias of the included studies using the Risk Of Bias In Non-randomised Studies - of Interventions. (ROBINS I) for non-randomised studies and Risk of Bias tool 2.0 for randomised studies.

Measures of treatment effect

Types of measurements of treatment effect (outcomes) that may be used:

1. Dichotomous data: we will use risk ratio for likely binary outcomes, prevalence ratio for some outcomes.

2. Continuous data: we will combine the results using the mean difference for measures using the same scale or the standardised mean difference where different scales have been used to evaluate the same outcome

Unit of analysis issues

We will consider the individual as the unit of analysis. We will separate the non-randomised from randomised studies if we have enough studies for meta-analysis.

For peer review only - http://bmjopen.bmj.com/site/about/guidelines.xhtml

Dealing with missing data

We will send two emails (one initial, one reminder) to the corresponding author to ask for any missing data or incompletely reported study details. We will check for consistency between studies and analyse each outcome.

Assessment of heterogeneity

We will assess the inconsistencies between studies using the I^2 statistic which gives the percentage of total variation across studies that is due to heterogeneity rather than chance. We will consider heterogeneity substantial if I^2 is over 50%. See below for planned subgroup analysis.

Assessment of reporting biases

If mismatches are identified between study protocols and reports, we will contact the trial authors to clarify the information. We plan to explore the impact of including such studies by conducting a sensitivity analysis. We will conduct a funnel plot asymmetry test if 10 or more trials are included.

Data synthesis

We will present the data separately for randomised and non-randomised studies. We will meta-analyse trials if the combination of data on outcomes is possible. We will use a random-effects model, independently of the heterogeneity identified. Forest plot graphics produced by RevMan 5.3 will illustrate the meta-analyses. If the combination of data is not possible, we will present a narrative analysis of individual studies. We will create a 'Summary of findings' table for the outcomes and we will present the quality of the body of evidence using the five GRADE assumptions (study limitations, consistency of effect, imprecision, indirectness and publication bias) [22].

Subgroup analysis and investigation of heterogeneity

We plan to explore the subsets or subgroups of countries/regions, for primary, secondary or tertiary care, type of mental health condition, gender, age, urban/rural area.

Sensitivity analysis

We will pool included studies to verify whether the impact of risk of bias affects the overall effect. We will explore which studies increased heterogeneity.

Ethics and dissemination

This systematic review will use published data and does not require ethics approval. However, this review is in preparation of a feasibility study that will examine how best to support the physical and mental health of Indigenous adolescents in Brazil. Ethics approval for the feasibility study was obtained from National Research Ethics Commission (CONEP), protocol CAAE: 89604318.0000.8030, analysis #3.100.358, from December of 2018.

Our results will also be presented at national and international conferences and will be made available to key decision-makers with authority for Indigenous health.

For peer review only

Quality of the evidence

Two review authors (AJG, CE) will independently rate the quality of the outcomes. We will use GRADE to rank the quality of the evidence using the Guideline Development Tool software[22] and also the guidelines provided in Chapter 11 of the Cochrane Handbook for Systematic Reviews of Interventions[23].

Patient and Public Involvement

The topic to be covered by the review comes from our empirical experience with the Indigenous communities in Brazil and from consultations with researchers in Indigenous health, and with practitioners and policy actors responsible for Indigenous health. The Indigenous people were not directly involved in the writing of this protocol. The results will be disseminated to indigenous communities, practitioners, and policy actors to aid the planning and provision of services. The authors are from Brazil and UK and do not self-identify as Indigenous.

CONCLUSION

This review will present the best-available evidence for decision making on suicide prevention programmes targeting Indigenous youth in the literature. It will provide in one single document all primary research on the topic with the quality assessment for each study with a ranking of the quality of evidence.

References

1. World Bank (2019) 'Understanding Poverty. Indigenous People', available online https://www.worldbank.org/en/topic/IndigenousPeoples (accessed July 17th, 2019).
2. Anderson I, Robson B, Connolly M, Al-Yaman F, Bjertness E, King A, Tynan M, Madden R, Bang A, Coimbra CE Jr, Pesantes MA, Amigo H, Andronov S, Armien B, Obando DA, Axelsson P, Bhatti ZS, Bhutta ZA, Bjerregaard P, Bjertness MB, Briceno-Leon R, Broderstad AR, Bustos P, Chongsuvivatwong V, Chu J, Deji, Gouda J, Harikumar R, Htay TT, Htet AS, Izugbara C, Kamaka M, King M, Kodavanti MR, Lara M, Laxmaiah A, Lema C, Taborda AM, Liabsuetrakul T, Lobanov A, Melhus M, Meshram I, Miranda JJ, Mu TT, Nagalla B, Nimmathota A, Popov AI, Poveda AM, Ram F, Reich H, Santos RV, Sein AA, Shekhar C, Sherpa LY, Skold P, Tano S, Tanywe A, Ugwu C, Ugwu F, Vapattanawong P, Wan X, Welch JR, Yang G, Yang Z, Yap L. Indigenous and tribal peoples' health (The Lancet-Lowitja Institute Global Collaboration): a population study. Lancet. 2016 Jul 9;388(10040):131-57.
3. Pollock NJ, Apok C, Concepcion T, Delgado RA Jr, Rasmus S, Chatwood S, Collins PY. Global goals and suicide prevention in the Circumpolar North. Indian J Psychiatry. 2020 Jan-Feb;62(1):7-14.
4. Lawson-Te Aho K, Liu JH. Indigenous suicide and colonization: The legacy of violence and the necessity of self-determination. International Journal of Conflict and Violence (IJCV). 2010 May 7;4(1):124-33.
5. Patton GC, Coffey C, Sawyer SM, Viner RM, Haller DM, Bose K, Vos T, Ferguson J, Mathers CD. Global patterns of mortality in young people: a systematic analysis of population health data. The lancet. 2009 Sep 12;374(9693):881-92.
6. Bilsen J. Suicide and Youth: Risk Factors. Front Psychiatry. 2018 Oct 30;9:540.

For peer review only - http://bmjopen.bmj.com/site/about/guidelines.xhtml

7. Hunter E, Harvey D. Indigenous suicide in Australia, New Zealand, Canada and the United States. Emergency Medicine. 2002 Mar;14(1):14-23.
8. Pollock NJ, Naicker K, Loro A, Mulay S, Colman I. Global incidence of suicide among Indigenous peoples: a systematic review. BMC medicine. 2018 Dec;16(1):145.
9. Harlow AF, Clough A. A systematic review of evaluated suicide prevention programs targeting Indigenous youth. Crisis. 2014.
10. Clifford AC, Doran CM, Tsey K. A systematic review of suicide prevention interventions targeting Indigenous peoples in Australia, United States, Canada and New Zealand. BMC public health. 2013 Dec;13(1):463.
11. Gracey M, King M. Indigenous health part 1: determinants and disease patterns. The Lancet. 2009 Jul 4;374(9683):65-75.
12. King M, Smith A, Gracey M. Indigenous health part 2: the underlying causes of the health gap. The lancet. 2009 Jul 4;374(9683):76-85.
13. United Nations Division for Sustainable Development (2018b) 'Indigenous Peoples and the Post-2015 Development Agenda', available online at https://www.un.org/development/desa/IndigenousPeoples/focus-areas/post-2015agenda.html (accessed May 8, 2019)
14. Welch V, Petticrew M, Tugwell P, Moher D, O'Neill J, Waters E, et al. (2012) PRISMA-Equity 2012 Extension: Reporting Guidelines for Systematic Reviews with a Focus on Health Equity. PLoS Med 9(10): e1001333.
15. UN Department of Economic and Social Aff airs. The concept of indigenous peoples. New York: United Nations, 2004.
16. UN Permanent Forum on Indigenous Issues. Who are indigenous peoples? http://www.un.org/esa/socdev/unpfi i/ documents/5session_factsheet1.pdf (accessed Feb 4, 2020).
17. Dickson JM, Cruise K, McCall CA, Taylor PJ. A Systematic Review of the Antecedents and Prevalence of Suicide, Self-Harm and Suicide Ideation in Australian Aboriginal and Torres Strait Islander Youth. Int J Environ Res Public Health. 2019 Aug 29;16(17):3154. doi: 10.3390/ijerph16173154. PMID: 31470580; PMCID: PMC6747538.
18. Bjorkenstam C, Kosidou K, Bjorkenstam E. Childhood adversity and risk of suicide: cohort study of 548 721 adolescents and young adults in Sweden. Bmj-Brit Med J. 2017;357.
19. Gaglio B, Shoup JA, Glasgow RE. The RE-AIM framework: a systematic review of use over time. Am J Public Health. 2013 Jun;103(6):e38-46.Eder, D.J. (2007). Bringing Navajo storytelling practices into schools: the importance of maintaining cultural integrity. Anthropol. Educ. Q., 38, 278-296.
20. Bachmann S. Epidemiology of Suicide and the Psychiatric Perspective. Int J Env Res Pub He. 2018;15(7).
21. Tobias, J. K., Richmond, C. A., & Luginaah, I. (2013). Community- based participatory research (CBPR) with indigenous communities: Producing respectful and reciprocal research. Journal of Empirical Research on Human Research Ethics, 8(2), 129–140.
22. GRADEpro Guideline Development Tool [Software] [Computer program]. McMaster University (developed by Evidence Prime, Inc.), Available from www.gradepro.org, 2015.
23. Schünemann HJ, Oxman AD, Higgins JPT, Vist GE, Glasziou P, Akl E, et al, on behalf of the Cochrane GRADEing Methods Group and the Cochrane Statistical Methods Group. Chapter 11: Completing 'Summary of findings' tables and grading

For peer review only

For peer review only - http://bmjopen.bmj.com/site/about/guidelines.xhtml

the confidence in or quality of the evidence. In: Higgins JPT, Churchill R, Chandler J, Cumpston MS, editor(s). Cochrane Handbook for Systematic Reviews of Interventions version 5.2.0 (updated June 2017). Cochrane, 2017 (available from www.training.cochrane.org/handbook).

Funding statement This work was supported by the Medical Research Council (MRC) grant number MR/R022739/1. Fundação de Apoio ao Desenvolvimento do Ensino, Ciência e Tecnologia do Estado de Mato Grosso do Sul, grant number 71/700070/2018

Authors' contributions AJG and CE wrote the protocol and approved the final version of the protocol; CP, PJ, PD, AV, JC, SH co-wrote the protocol, critically reviewed and approved the final version.

Competing interests: The authors declare no competing interests

For peer review only

Reporting checklist for protocol of a systematic review.

Based on the PRISMA-P guidelines.

Instructions to authors

Complete this checklist by entering the page numbers from your manuscript where readers will find each of the items listed below.

Your article may not currently address all the items on the checklist. Please modify your text to include the missing information. If you are certain that an item does not apply, please write "n/a" and provide a short explanation.

Upload your completed checklist as an extra file when you submit to a journal.

In your methods section, say that you used the PRISMA-Preporting guidelines, and cite them as:

Moher D, Shamseer L, Clarke M, Ghersi D, Liberati A, Petticrew M, Shekelle P, Stewart LA. Preferred Reporting Items for Systematic Review and Meta-Analysis Protocols (PRISMA-P) 2015 statement. Syst Rev. 2015;4(1):1.

		Reporting Item	Page Number
**Title**			
Identification	#1a	Identify the report as a protocol of a systematic review	1,2
Update	#1b	If the protocol is for an update of a previous systematic review, identify as such	N/A
**Registration**			
	#2	If registered, provide the name of the registry (such as PROSPERO) and registration number	2
**Authors**			
Contact	#3a	Provide name, institutional affiliation, e-mail address of all protocol authors; provide physical mailing address of corresponding author	1
Contribution	#3b	Describe contributions of protocol authors and identify the guarantor of the review	1,9

For peer review only

**Amendments**			
	#4	If the protocol represents an amendment of a previously completed or published protocol, identify as such and list changes; otherwise, state plan for documenting important protocol amendments	
**Support**			
Sources	#5a	Indicate sources of financial or other support for the review	9
Sponsor	#5b	Provide name for the review funder and / or sponsor	9
Role of sponsor or funder	#5c	Describe roles of funder(s), sponsor(s), and / or institution(s), if any, in developing the protocol	9
**Introduction**			
Rationale	#6	Describe the rationale for the review in the context of what is already known	3,4
Objectives	#7	Provide an explicit statement of the question(s) the review will address with reference to participants, interventions, comparators, and outcomes (PICO)	4
**Methods**			
Eligibility criteria	#8	Specify the study characteristics (such as PICO, study design, setting, time frame) and report characteristics (such as years considered, language, publication status) to be used as criteria for eligibility for the review	4
Information sources	#9	Describe all intended information sources (such as electronic databases, contact with study authors, trial registers or other grey literature sources) with planned dates of coverage	5
Search strategy	#10	Present draft of search strategy to be used for at least one electronic database, including planned limits, such that it could be repeated	Appendix
Study records - data management	#11a	Describe the mechanism(s) that will be used to manage records and data throughout the review	6
Study records - selection process	#11b	State the process that will be used for selecting studies (such as two independent reviewers) through each phase of the review (that is, screening, eligibility and inclusion in meta-analysis)	6

For peer review only

Study records - data collection process	#11c	Describe planned method of extracting data from reports (such as piloting forms, done independently, in duplicate), any processes for obtaining and confirming data from investigators	6
Data items	#12	List and define all variables for which data will be sought (such as PICO items, funding sources), any pre-planned data assumptions and simplifications	6
Outcomes and prioritization	#13	List and define all outcomes for which data will be sought, including prioritization of main and additional outcomes, with rationale	4,5
Risk of bias in individual studies	#14	Describe anticipated methods for assessing risk of bias of individual studies, including whether this will be done at the outcome or study level, or both; state how this information will be used in data synthesis	7
Data synthesis	#15a	Describe criteria under which study data will be quantitatively synthesised	7
Data synthesis	#15b	If data are appropriate for quantitative synthesis, describe planned summary measures, methods of handling data and methods of combining data from studies, including any planned exploration of consistency (such as I2, Kendall's τ)	
Data synthesis	#15c	Describe any proposed additional analyses (such as sensitivity or subgroup analyses, meta-regression)	
Data synthesis	#15d	If quantitative synthesis is not appropriate, describe the type of summary planned	7
Meta-bias(es)	#16	Specify any planned assessment of meta-bias(es) (such as publication bias across studies, selective reporting within studies)	
Confidence in cumulative evidence	#17	Describe how the strength of the body of evidence will be assessed (such as GRADE)	8

None The PRISMA-P checklist is distributed under the terms of the Creative Commons Attribution License CC-BY 4.0. This checklist can be completed online using https://www.goodreports.org/, a tool made by the EQUATOR Network in collaboration with Penelope.ai

For peer review only
